# Chicken white egg chemerin as a tool for genetic selection for egg weight and hen fertility

**DOI:** 10.3389/fphys.2022.1012212

**Published:** 2022-09-13

**Authors:** Ophélie Bernardi, Maxime Reverchon, Anthony Estienne, Yannick Baumard, Christelle Ramé, Adeline Brossaud, Yves Combarnous, Pascal Froment, Joëlle Dupont

**Affiliations:** ^1^ Centre National de la Recherche Scientifique, Institut Français du Cheval et de l’Equitation, Institut National de Recherche pour l’Agriculture, l’Alimentation et l’Environnement (INRAE), Université de Tours, Physiologie de la Reproduction et des Comportements, UMR85, Paris, France; ^2^ SYSAAF-Syndicat des Sélectionneurs Avicoles et Aquacoles Français, Centre INRA Val de Loire, France; ^3^ INRAE—Unité Expérimentale Pôle D’expérimentation Avicole de Tours, France

**Keywords:** hen, egg white, chemerin, fertility, egg performance

## Abstract

Embryo mortality rate, which can reach up to 40% in avian species, is a major issue for breeding. It is therefore important to identify new embryo development biomarkers for genetic selection to improve reproductive performances. We have recently shown that chemerin is expressed in the oviductal hen magnum, accumulates in egg white, is correlated with embryo survival and could thus be used as a molecular marker of embryo development. Eggs from seven hen breeds (*n* = 70) were collected during five successive days at the end of the laying period. After weighing eggs, yolk and albumen, an egg white sample from each egg was collected and a blood sample was taken from each hen. Chemerin concentrations in albumen and blood samples were measured by a specific home made ELISA assay. Hen’s plasma and egg’s albumen chemerin levels were found to be correlated with reproductive parameters such as fecundity, fertility, embryo mortality, hatchability and laying rates. The inter-hen chemerin level variability in albumen was higher than intra-hen except for one breed (R+). We observed significantly different levels of chemerin in egg white between breeds. However, chemerin concentrations in egg white were not significantly associated to variations of hen plasma chemerin levels. Interestingly, we observed negative correlations between albumen chemerin concentrations and egg weight (*r* = −0.43, *p* = 0.001), between albumen weight (*r* = −0.40, *p* = 0.002), and between yolk weight (*r* = −0.28, *p* = 0.03). We also showed negative correlations between egg white chemerin concentrations and fecundity (*r* = −0.32, *p* = 0.011) and fertility (*r* = −0.27, *p* = 0.04) whereas no significant correlation was observed with the laying rate. Taken together, these results suggest that egg white chemerin concentration might be a good biomarker for genetic selection for egg weight and fertility in hens, provided these data are confirmed on a larger scale.

## Introduction

One of the breeding companies’ goals is the production of viable and robust one day-old chicks to renew their reproductive flock. However, embryo and chick mortality rates during the first week of their life are major issues for profitability and competitiveness in poultry industry. This early mortality rate is an important and often used index to assess chick quality and it is also a welfare indicator (Tona et al., 2005). In addition to environmental factors, other parameters such as hen and egg production type, storage of eggs, albumen and egg quality affect the embryo development, hatchability and chick quality (Peebles et al., 2001; Schmidt et al., 2009; Nasri et al., 2020; Özlü et al., 2021). New tools are necessary to improve the quality of embryo development and the eggs hatchability. Indeed, the identification of new regulators/biomarkers of embryo development is a potential way to improve production performances at all steps of chicken breeding (laying, fertility and hatchability rates and the robustness of chicks). Nutrition and energy metabolism are well known to influence reproduction in all vertebrates. In broiler chicken, the rapid growth and the fattening are often associated with undesirable reproductive capacities such as a high sexual precocity, a loss of the follicular hierarchy, multiple ovulations and a decrease of egg production, fertility and hatchability (Chen et al., 2006; Briere et al., 2011; Zhang et al., 2018). Some variations in nutritional status could impact the reproductive system through changes in plasma metabolic protein secretions (Briere et al., 2011). Indeed, metabolic tissues synthesize and release protein and peptides hormones named adipokines that are involved in the regulation of various physiological functions including reproduction in mammals but also in birds (Ahima and Flier, 2000; Zhang et al., 2018; Estienne et al., 2020; Bernardi et al., 2021).

Chemerin was discovered in 1997 as a novel retinoid-responsive gene in human skin (Nagpal et al., 1997). Later, chemerin (18 kDa), also named TIG2 (tazarotene-induced gene 2) or RARRES2 (retinoic acid receptor responder 2), was identified as an adipokine mainly secreted by adipose tissue and liver. In mammals, chemerin is expressed in several other tissues such as pancreas, placenta, skin, kidneys, intestines and gonads (Mattern et al., 2014). After several successive cleavages, the active form of chemerin is able to bind three G-coupled receptors with seven transmembrane domains named CMKLR1 (Chemokine like receptor 1), GPR1 (G protein receptor 1) and CCRL2 (C-C chemokine receptor-like 2) (Yoshimura and Oppenheim, 2011). Chemerin is involved in many physiological processes such as the regulation of immune system and adipogenesis, angiogenesis, reproductive functions and metabolic disorders (Bernardi et al., 2021). Bozaoglu et al. showed plasma chemerin levels are positively associated with body fat, plasma glucose and triglyceride and lipid metabolism in humans (Bozaoglu et al., 2007). However, in contrary to mammals, plasma chemerin levels are negatively correlated with fattening in turkeys (Diot et al., 2015) and chickens (Mellouk et al., 2018d). Mellouk et al. studied the expression profiles of various adipokines in metabolic tissue during the chicken embryo development and they showed that the chemerin amount was specifically increased at hatching in various metabolic tissues, and this was associated with an increase in the weight of the embryo (Mellouk et al., 2018c). Concerning reproductive functions, many studies demonstrated the expression of chemerin and its receptors in ovary including granulosa and theca cells, corpus luteum and oocytes in different mammals including humans. These studies showed that chemerin inhibits Insulin-like Growth Factor 1 (IGF-1)-induced steroidogenesis in primary granulosa cells (Reverchon et al., 2012, 2014). Chemerin hormone and receptor are expressed by granulosa and theca cells in both broiler hens (Mellouk et al., 2018a) and turkeys (Diot et al., 2015) but at different levels. Moreover, proteomic analyzes have identified chemerin within the albumen and perivitelline membranes of chicken eggs (Mann, 2007, 2008). Our laboratory recently demonstrated that chemerin is highly present in the albumen compared to the yolk and plasma (Estienne et al., 2022). Moreover in the oviduct of hens, chemerin and its receptors are more expressed in the magnum, where the egg white is formed (Estienne et al., 2022). Also, a chemerin system is present in extraembryonic membranes such as allantoic and amniotic membranes and fluids suggesting a passage of chemerin from albumen to the extraembryonic membranes (Estienne et al., 2022). Mellouk et al. showed that plasma chemerin concentrations were negatively correlated with egg hatchability in chicken (Mellouk et al., 2018b). In addition, we also demonstrated an increase of embryo mortality after *in ovo* injections of anti-chemerin and anti-CMKLR1 antibodies in egg white (Estienne et al., 2022). Thus, our working hypothesis is that chemerin in egg white is involved in the embryo development in chicken and could be a new reproductive trait for genetic selection.

The aims of the present study were 1. To investigate the egg white and plasma concentrations of chemerin using a new home-made chicken chemerin ELISA, 2. To compare them in individual laying hens, and in successive eggs from the same hen to evaluate the intra and inter hen coefficient of variation and 3. To determine potential relationships between egg white chemerin concentration and hen egg performance and fertility parameters.

## Material and methods

### Ethical issues

All the zootechnical parameters (total egg laying, incubated eggs, unfertilized eggs, eggs mortality and hatched chicks) were routinely collected by the Experimental Unit “Pôle Expérimental Avicole de Tours” UEPEAT (INRAE, Nouzilly, France, doi: https://doi.org/10.15454/1.5572326250887292E12) for the monitoring of the breeding. Plasma and tissues were collected during meat processing as abattoir by-products by highly qualified and experienced laboratory staff. Thus, according to the ethical issues for the protection of animals, this project does not require the consent of the competent ethics committee for animal experiments. The UEPEAT experimental unit is registered by the Ministry of Agriculture with the license number D-37-175-1 for animal experimentation. All experiments were performed in accordance with the European Communities Council Directive 2010/63/UE. Human plasma was collected from patients (*n* = 3) undergoing bariatric surgery and included in the prospective monocentric METABOSE cohort (Nutrition Department, CHU Tours) following patient written consent and after local ethical committee agreement (CNIL n° 18254562). Mice plasma was collected from three control adult animals included in a protocol approved by an ethics committee (Comité d’Ethique en Expérimentation Animale Val de Loire, CEEA VdL, protocol reference number 2021032516245453.V2-30673).

### Animals and samples collection

**FIGURE 1:**
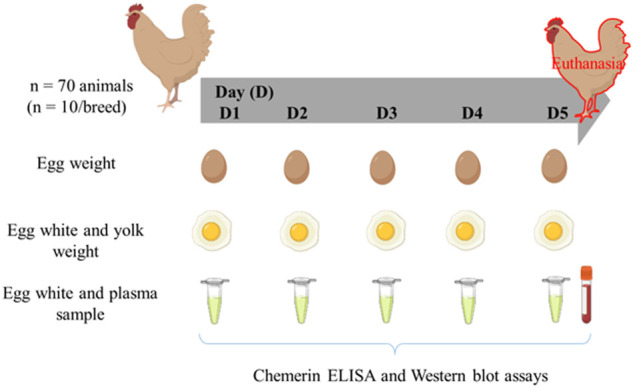
Experimental design. Seventy fasted hens (54 weeks-old, end of laying) from seven Rhode Island breeds (*n* = 10 hen per breed) were used. During five days of laying, eggs were successively collected in order to weigh the egg, egg white (albumen) and yolk individually. For each egg, an albumen sample was stored at −20°C until use. At the end of the protocol, all hens were euthanized by electrical stunning and bled out for blood sample collection.

Seven breeds composed of 6–10 hens (54 weeks-old, end of laying) were reared at UEPEAT according to the conventional breeding conditions. The peculiarities of each breed are summarized in Table 1. The number of animals used for each breed is indicated in Table 2. During five successive days of laying, eggs were collected every day in order to weigh the egg, egg white (albumen) and yolk individually. For each egg, an albumen sample was collected and stored at −20°C until use. At the end of the protocol, all overnight fasted hens were euthanized by electrical stuning and bled out for blood sample collection, as recommended by the ethical committee. Blood samples were centrifuged (5000 g for 10 min at 4°C) and plasma samples stored at −20°C until use. The experimental design is summarized in Figure 1. Nine hens out of 70 were eliminated from the protocol because they laid less than three eggs that’s why *n* = 6 to 10 hens dependent on the breed.

**TABLE 1:** Peculiarities and phenotypes of the breeds used for experimental design.

Breed	Pecularities	References
R-/R+ (Rhode Island Red)	Selected for low and high residual feed consumption and metabolism and reproduction performances	Tixier-Boichard et al. (1995)
FAYOUMI (Local breed)	Origin Egypt and resistance to disease in particular coccidiosis	(Hamet et al., 1982; Mérat et al., 1983)
OD (Leghorn)	Multiple ovulations	Lowry et al. (1979)
GAVORA (Leghorn)	Absence of endogenous viral genes	Gavora et al. (1989)
	High egg production	
CHEPTEL1 (Local breed)	Origin of variant Pea comb	Chang et al. (2007)
DwNa (INRAE breed)	DWARF gene for metabolism and laying performances	(Chen and Tixier-Boichard, 2003a; 2003b)

**TABLE 2:** Egg performances of different breed. Data are shown as the mean ± SEM; *n* = 6–10 animals per breed. Groups showing different letters are significantly different (*p < 0.05*). The laying rate was determined during 20 successive weeks in 21 weeks-old animals from different breeds.

Breed		Laying (%)	Egg weight (g)	Albumen weight (g)	Yolk weight (g)	Ratio albumen/yolk	Ratio egg/albumen
R-	*n* = 9	85.39 ± 1.18^ab^	54.65 ± 0.98^bc^	30.80 ± 0.64^b^	16.31 ± 0.33^ab^	1.89 ± 0.05^bc^	1.78 ± 0.02^b^
R+	*n* = 9	90.12 ± 1.11^b^	52.16 ± 1.21^b^	28.53 ± 0.91^ab^	16.48 ± 0.38^ab^	1.73 ± 0.05^ab^	1.83 ± 0.02^ab^
FAYOUMI	*n* = 10	79.97 ± 1.76^a^	43.89 ± 1.04^a^	21.75 ± 0.63^a^	14.80 ± 0.49^a^	1.48 ± 0.05^a^	2.02 ± 0.04^a^
OD	*n* = 7	91.61 ± 4.02^b^	60.68 ± 1.38^c^	33.85 ± 0.93^b^	18.04 ± 0.61^b^	1.88 ± 0.05^bc^	1.80 ± 0.02^b^
GAVORA	*n* = 10	86.80 ± 2.23^ab^	58.13 ± 0.93^bc^	32.66 ± 0.52^b^	16.80 ± 0.33^ab^	1.95 ± 0.03^c^	1.78 ± 0.01^b^
CHEPTEL1	*n* = 10	83.36 ± 2.30^ab^	54.55 ± 1.06^bc^	29.66 ± 0.78^b^	16.96 ± 0.33^b^	1.75 ± 0.04 ^abc^	1.84 ± 0.02^ab^
DwNa	*n* = 6	89.61 ± 2.21^ab^	55.32 ± 1.33^bc^	29.69 ± 0.95^ab^	17.39 ± 0.42^b^	1.71 ± 0.03^abc^	1.87 ± 0.02^ab^
*p value*		0.004	<0.0001	<0.0001	0.002	<0.0001	<0.0001

### Reproductive parameters

The reproductive parameters of the animals (6-10 animals in each breed) were determined at 21 week-old during 20 successive weeks (Table 3). For each breed, the semen of several roosters was collected and pooled to form a single sample. The hens were artificially inseminated with 2 × 10^8^ spermatozoa from the pool and eggs were collected and counted daily for two weeks following the artificial insemination and then artificially incubated. The number of unfertilized eggs, and early (EEM) and late (LEM) embryonic mortality were evaluated by breaking eggs and candling on the 7th (EEM) and 14th day of incubation (LEM).

**TABLE 3:** Reproductive parameters of the different breed. Data are shown as the mean ± SEM; *n* = 6–10 animals per breed. Groups showing different letters are significantly different (*p < 0.05*).

Breed	Number of animals (n)	Fecundity (%)	Fertility (%)	EEM (%)	LEM (%)
R-	9	77.16 ± 10.63	68.94 ± 9.19	4.90 ± 2.01	3.89 ± 2.30
R+	9	74.73 ± 8.40	60.37 ± 10.82	16.88 ± 8.39b	3.46 ± 2.40
FAYOUMI	10	87.10 ± 4.90	85.43 ± 5.59	0a	2.22 ± 2.22
OD	7	88.78 ± 7.61	80.12 ± 8.02	5.77 ± 2.27	4.52 ± 3.54
GAVORA	10	92.33 ± 3.17	84.95 ± 4.15	4.81 ± 1.71	3.39 ± 1.95
CHEPTEL1	10	83.06 ± 5.91	78.45 ± 6.19	5.25 ± 2.34	0.77 ± 0.77
DwNa	6	85.48 ± 6.04	79.69 ± 5.20	3.75 ± 2.60	2.67 ± 1.69
*p value*		0.56	0.28	0.10	0.84

For each hen, the different performances and reproductive parameters (laying, fecundity, fertility, EEM, LEM and hatchability percentage) were calculated using the following formulae:

$$\%\;laying=\frac{number\;of\;eggs\;laid}{number\;of\;laying\;days}\;x\;100$$ (1)

$$\%\;fecundity=\frac{\left(number\;of\;incubated\;eggs-unfertilized\;eggs\right)}{number\;of\;incubated\;eggs}\;x\;100$$

$$\%\;fertility=\frac{\left(number\;of\;incubated\;eggs-unfertilized\;eggs-number\;of\;egg\;mortality\right)}{number\;of\;incubated\;eggs}\;x\;100$$

$$\%\;EEM=\frac{number\;of\;EEM}{\left(number\;of\;incubated\;eggs-unfertilized\;eggs\right)}\;x\;100$$

$$\%\;LEM=\frac{number\;of\;LEM}{\left(number\;of\;incubated\;eggs-unfertilized\;eggs+number\;of\;EEM\right)}\;x\;100$$

$$\%\;hatchability=\frac{number\;of\;hatched\;chicks\;}{number\;of\;fertile\;eggs\;after\;14\;days\;of\;incubation}\;x\;100$$

### Production of recombinant chicken chemerin and monoclonal chicken chemerin antibody

The recombinant chicken chemerin protein (full length, rRARRES2) was obtained from the *Gallus gallus* sequence (NM_001277476.1), produced in *Escherichia coli* and purified by a chromatography column-based on His-Tag under denaturing conditions (Agro-Bio, La Ferté Saint Aubin, France) (Estienne et al., 2020). Monoclonal chicken chemerin antibodies were produced by AgroBio (La Ferté Saint Aubin, France), and their specificity was tested as previously described (Mellouk et al., 2018a, 2018c; Estienne et al., 2020, 2021).

### Detection and quantification of chemerin by western blot analysis

Egg whites were lysed using an Ultraturax (Invitrogen™ by Life Technologies™, Villebon sur Yvette, France) in lysis buffer 50% (vol/vol, Tris 1 M (pH 7.4), NaCl 0.15 M, EDTA 1.3 mM, EGTA 1 mM, VO43−23 mM, NaF 0.1 M, NH2PO41%, Triton 0.5%). The protein concentration of lysates was measured using the bicinchoninic acid (BCA) protein assay (Interchim, Montluçon, France). Egg white (80 µg) were mixed with Laemmli buffer 5 X and proteins were denatured for 5 min by heating at 95°C. Proteins were loaded on an electrophoresis sodium dodecyl sulfate-polyacrylamide gel (12% for high protein weight (110-20 kDa) or 15% for low protein weight (<20 kDa)) and then proteins were transferred to a nitrocellulose membrane. Membranes were blocked with Tris-Buffered Saline Tween buffer containing 0.05% of Tween 20 and 5% of milk for 30 min at room temperature. Membranes were incubated overnight at 4°C with the appropriate primary antibody (anti-chemerin antibody). Then, membranes were incubated 90 min at room temperature with a Horse Radish Peroxidase-conjugated anti-mouse IgG. Chemerin protein was detected by enhanced chemiluminescence (ECL, Western Lightning Plus-ECL, Perkin Elmer, Villebon-sur-Yvette, France) with a G-box SynGene (Ozyme, St Quentin en Yvelines, France) and the GeneSnap software (Ozyme, St Quentin en Yvelines, France). Then, protein amounts were quantified with GeneTools software. The results were expressed as the intensity signal in arbitrary units after normalization of chemerin protein signals with the total protein amount as evaluated by Ponceau-red staining. Albumen of eggs from different avian species such as Galliforms (chicken *Gallus gallus*, turkey *Meleagris gallopavo*, pheasant *Phasianus colchicus*, quail *Coturnix japonica,* guinea fowl *Numida meleagris* and red-legged partridge *Alectoris rufa*), Anseriforms (ducks *Cairina moschata* and *Anas platyrhynchos* and goose *Anser anser*) and Colombiform (pigeon *Columba livia*) species were tested. All these eggs were provided by different breeders from Région Centre Val de Loire (France).

### Development of a sandwich ELISA for chicken chemerin

Microtiter plates (NUNC Maxisorb, ThermoFisher Scientific, Les Ulis, France) were coated with bovine serum albumin (100 mM NaHCO3 pH 9.6) at 4°C for 16 h. After blocking of free binding sites with 200 μL of assay buffer containing 0.2% BSA and 0.01% Tween 20 in PBS pH 7.3 at RT for 1 h, the plates were washed 3 times with PBS containing 0.01% Tween 20, filled with 200 μL of assay buffer. Then, serial 2-fold dilutions of the chicken recombinant chemerin (256–2 ng/ml) were prepared in the assay buffer to generate the calibration curve. For the assay, plates were decanted, and 100 μL of reference recombinant chicken chemerin or prediluted white egg in lysis buffer (Tris 1 M (pH 7.4), NaCl 0.15 M, EDTA 1.3 mM, EGTA 1 mM, VO43−23 mM, NaF 0.1 M, NH2PO41%, Triton 0.5%) (1/2) or pure plasma were pipetted in duplicate into the specified wells and then incubated for 3 h at room temperature. After 3 washings, one hundred microliters of the monoclonal chicken chemerin antibody (used at final concentration of 100 ng/ml) was added and the plate was incubated for 1 h at RT. After 3 washings, 100 μL of peroxidase-conjugated secondary antibody (1/2000; Sigma; A1949) was added and incubated for 1 h at RT. After 3 washings, the TMB substrate solution (100 μL) was added and incubated for 30 min at 25°C in the dark. The reaction was stopped with 50 μL of 1 M H2SO4, and optical density was determined at 450 nm with a microtiter plate reader (Tecan, Magellan, Männedorf, Switzerland). A standard curve was drawn for the determination of chemerin levels. The accuracy of the ELISA was determined by showing parallelism of serial dilutions of chicken serum and egg white samples and the standard curve. No cross-reactivity was shown for human, bovine, ovine and goat serum samples. The mean recovery of 4 different concentrations of recombinant chicken chemerin spiked into a serum and egg white sample determined in 4 different dilutions was 118 ± 9.2% and 116% ± 8.5, respectively. The intra-assay and interassay variation were determined by repeated measurement (*n* = 10) of 2 different serum and egg white samples at 2 different dilutions (*n* = 4) within one assay plate and across different assays (*n* = 8). The mean intra-assay CV was 6 and 8%, the inter-assay CV was 10 and 11% for blood plasma and egg white samples, respectively.

### Alignment of nucleic and proteic RARRES 2 sequence of various avian species

The gene and protein sequences of *RARRES2* of different avian species were downloaded from the National Centre for Biotechnology Information (NCBI http://www.ncbi.nlm.nih.gov) under GenBank accession number. Multiple RARRES2 sequences of different avian species were aligned using Clustal Omega to check their percent homology and identity matrix for *RARRES2* gene and protein of avian species compared to *RARRES*2 chicken.

### Statistical analysis

The GraphPad Prism^®^ software (version 6) was used for all analyses. All data are represented as means ± standard error of mean (SEM) with a level of significance less than 0.05 (*p < 0.05*). One-way ANOVA and multiple comparisons were used to compare reproductive parameters, chemerin concentration in albumen and blood among the different breeds. A Pearson test was used to analyse correlations. The correlation was noted “*r*” and the *p* value was considered significant if *p* value <0.05. Different letters indicate significant differences (*p < 0.05*).

## Results

### Laying performance and reproductive parameters of chicken breeds

As described in Table 2, we used seven breeds of hen (*n* = 61) that have significant difference on laying rate, egg weight and egg white and yolk weights. Overall, Fayoumi hens lay significantly fewer eggs than R+ and OD animals (Table 2). The egg weights were lower in Fayoumi hens compared to the other breeds (Table 2), whereas the reproductive parameters, i.e. the percentages of fecundity, fertility, EEM, LEM and hatchability were similar in the seven breeds under study (Table 3).

### Chemerin in hen plasma and egg white

**FIGURE 2:**
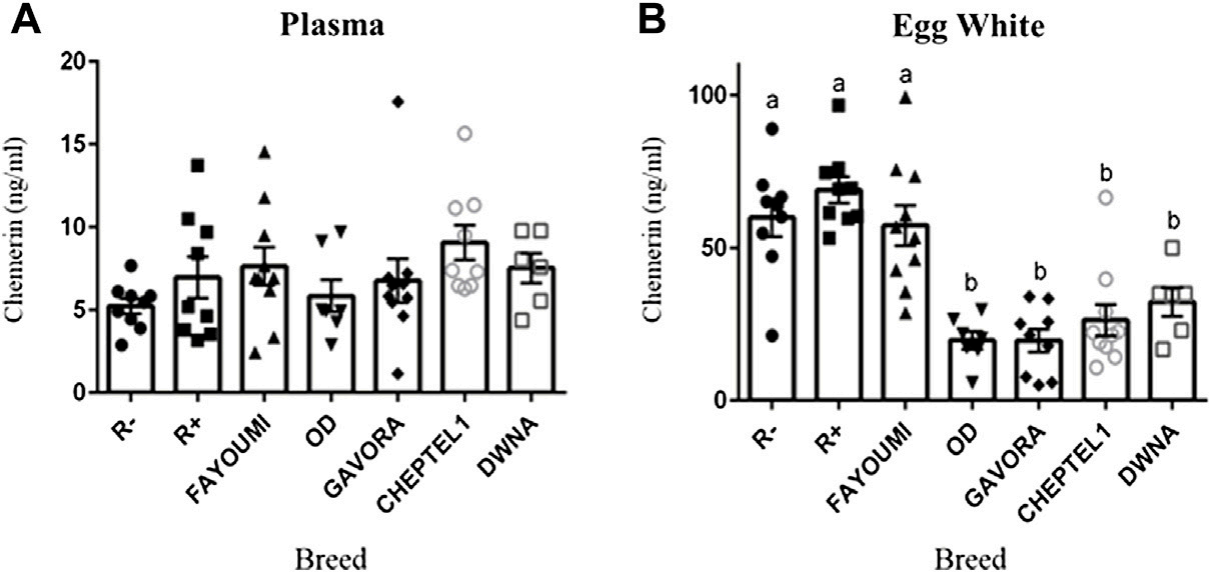
Concentration of chemerin in plasma **(A)** and egg white **(B)** in each breed of hen as determined by ELISA assay. Concentration of chemerin in plasma **(A)** and in egg white from eggs **(B)** was determined by ELISA assay in seventy 54-old hens from seven different breeds. Data are shown as the mean ± SEM; *n* = 6–10 animals per breed. Groups showing different letters are significantly different (*p* < 0.05).

As shown in Figure 2A, hen plasma chemerin concentrations were similar (*p= 0.261*), whereas they were very different in egg whites from the seven breeds. In addition, as shown in Figure 2B, chemerin concentrations were up to 5 to 10 fold higher in egg white as compared to their mother’s plasma. Chemerin concentration in egg white was about two-fold higher in R-, R+ and Fayoumi breeds than in OD, Gavora, Cheptel1 and DwNa breeds (*p < 0.0001*) (Figure 2B). The intra-hen variability coefficient was determined from chemerin concentrations in albumen of each egg laid during five successive days, whereas the inter-hen variability coefficient was calculated from chemerin concentrations in egg white for all hens of each breed. Data showed a lower intra-hen variability of chemerin concentration compared with the inter-hen variability except for R+ breed (Table 4). Most importantly, in the seven breeds of hen (*n* = 61 animals), no correlation was observed between chemerin concentrations in egg white and in their mother’s plasma (Table 5).

**TABLE 4:** Concentration of chemerin in biological fluids (plasma and albumen) and their variability inter-hen and intra-hen. Data are shown as the mean ± SEM; *n* = 6–10 animals per breed. Groups showing different letters are significantly different (*p < 0.05*).

Breed	Number of animals (n)	Chemerin plasma (ng/ml)	Variability inter-hen (%)	Chemerin albumen (ng/ml)	Variability inter-hen (%)	Variability intra-hen (%)
R-	9	5.23 ± 0.46	26.50	59.86 ± 6.15^a^	30.81	29.06
R+	9	6.97 ± 1.25	53.79	68.96 ± 4.27^a^	18.56	29.20
FAYOUMI	10	7.62 ± 1.14	47.41	57.29 ± 6.68^a^	36.86	26.61
OD	7	5.83 ± 0.96	43.79	19.74 ± 2.92^b^	39.17	22.51
GAVORA	10	6.76 ± 1.32	61.74	19.63 ± 3.56^b^	57.30	28.31
CHEPTEL1	10	9.07 ± 1.00	34.90	26.39 ± 5.13^b^	61.44	27.95
DwNa	6	7.52 ± 0.90	29.31	32.32 ± 4.70^b^	35.61	27.04
*p value*		0.26		<0.0001		

**TABLE 5:** Pearson correlation coefficient (r) calculated between chemerin concentration in plasma and egg white with egg performances and reproductive parameters in 7 breeds of hen (*n* = 61). The correlation noted « r » and *p*-value was significant if *p* < 0.05. NS, indicated no difference significant.

*r*	Egg weight	Albumen weight	Yolk weight	Laying	Fecundity	Fertility	EEM	LEM	Hatchability	Chemerin plasma	Chemerin albumen
*p value*											
Chemerin plasma	NS	NS	NS	NS	NS	NS	NS	NS	NS		NS
Chemerin albumen	−0.43	−0.39	−0.28	NS	−0.32	−0.27	NS	NS	NS	NS	
	0.001	0.002	0.03		0.01	0.03					

### Comparison of chemerin concentration in hen egg white measured by ELISA and western-blot analysis

**FIGURE 3:**
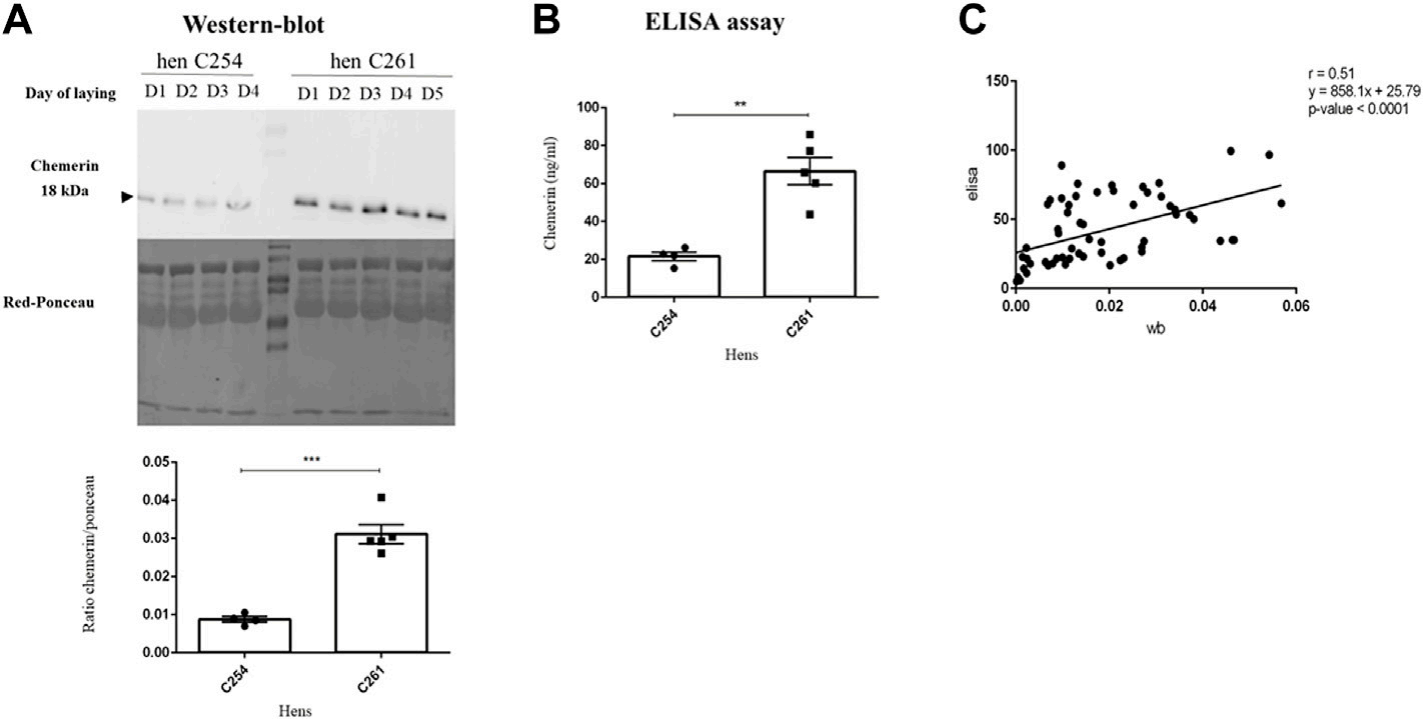
Concentration of chemerin in egg white by two methods of analysis. **(A)** Protein abundance of chemerin detected by western blotting within 3 or 4 or 5 egg whites laid successively in two hens named C254 and C261, respectively (*n* = 4–5). **(B)** Chemerin concentration determined by ELISA assay within the same egg whites samples as **(A)**. Data are shown as the mean ± SEM; *n* = 4 to 5 samples per animal. Significant differences are indicated by *p* < 0.01 ** and ***p < 0.001. **(C)** Sixty-one egg white samples from eggs laid by different hens were collected and analysed by both ELISA and Western-blot assays. Correlation between chemerin concentration within albumen obtained by western blotting and ELISA assay (*n* = 61) is shown.

Samples of the hens’s albumen were analyzed by two methods: Western-blot and ELISA. As shown in Figure 3A, the amount of egg white chemerin as determined by western-blot analysis in 4 or 5 successive eggs showed a three-fold difference between two hens (C254 *vs*. C261 hen). This difference was similar between egg white chemerin concentrations measured by ELISA (Figure 3B). These data were confirmed for six hens. We also determined correlation between these two methods (ELISA *vs.* Western-blot) by using egg white samples from hens of seven breeds (*n* = 61 with only one value for each hen). In this case, the correlation analysis confirmed the comparability of the results from both methods (*p < 0.0001*); r was 0.51 when individual data (egg white from different hens) were used (Figure 3C).

### Chemerin concentration in plasma and egg white associated with performance parameters

We next analyzed a potential link between chemerin concentrations in egg white and plasma with egg performance and reproductive parameters with Pearson correlations by using data from the seven breeds of hen (*n* = 61 animals). As shown in Table 5, chemerin concentration in egg white was negatively correlated with egg weight (*r = −0.43, p = 0.001*), albumen weight (*r = - 0.39, p = 0.002*) and yolk weight (*r = - 0.28, p = 0.029*). Concerning reproductive parameters, we also evidenced negative correlations between chemerin concentrations in egg white and hen fecundity (*r = - 0.32, p = 0.01*) and fertility (*r = - 0.27, p = 0.037*) (Table 5). However, no correlation was observed between plasma chemerin levels and egg performances or reproductive parameters (Table 5).

### Chemerin presence in egg white from other avian species

**FIGURE 4:**
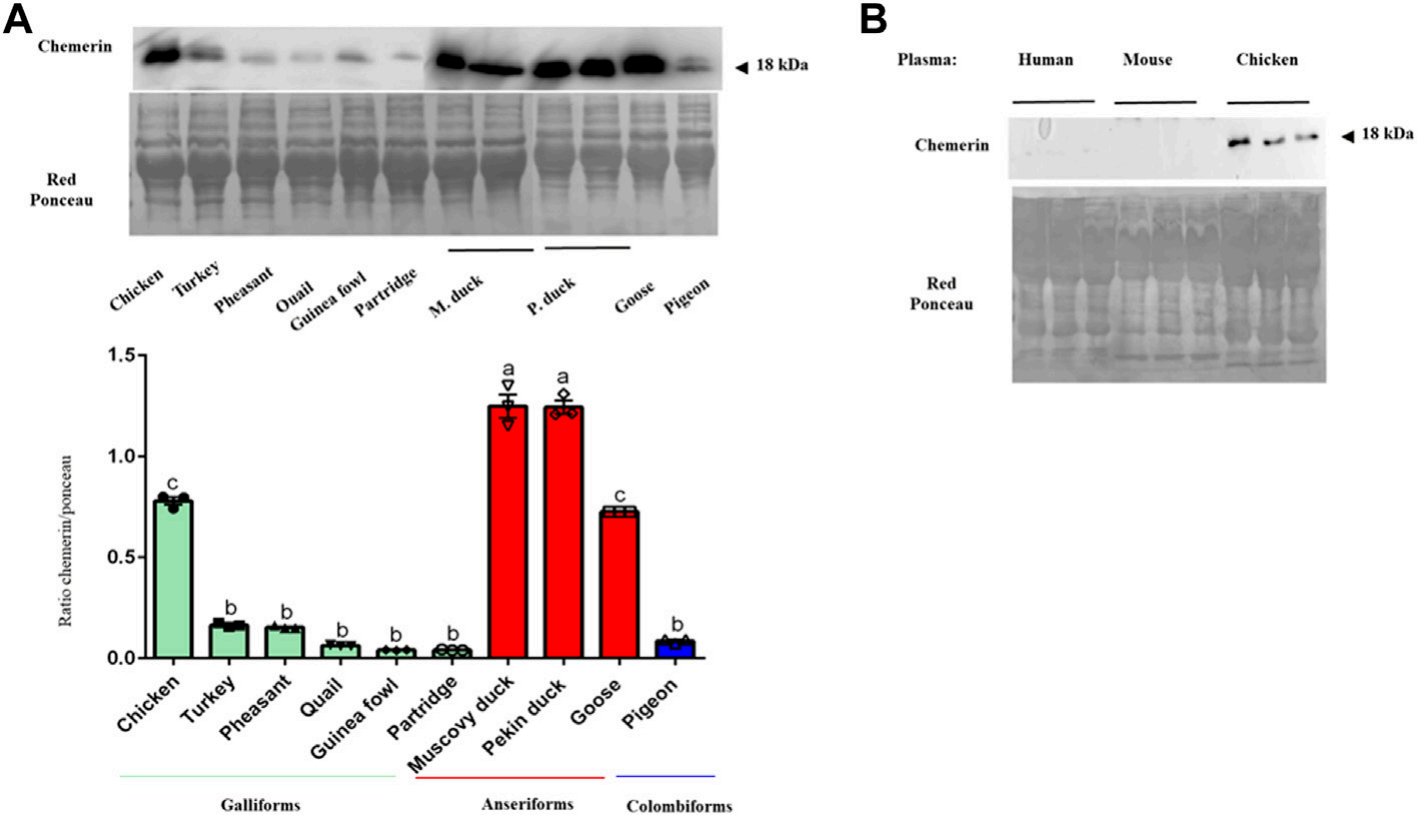
Abundance of chemerin in egg white of different avian species. **(A)** Protein amount of chemerin in egg white from egg of different avian species was detected by western blotting. Data are shown as the mean ± SEM; *n* = 3 egg white samples from different animals of various avian species. Groups showing different letters are significantly different (*p* < 0.05). **(B)** Chemerin protein expression in human, mouse and chicken blood plasma samples (*n* = 3 for each species). All plasma samples contained equal amounts of proteins, as confirmed by staining the nitrocellulose membrane with Red Ponceau.

Egg white samples from different avian species were analyzed by Western blot to detect chemerin by using our monoclonal chicken chemerin antibody. As shown in Figure 4, we found the presence of chemerin in egg white in Galliforms including inter alia chicken, turkey, quail, guinea fowl, red partridge and pheasant with a higher amount in chicken. Chemerin in egg white was also detected in Anseriforms such as muscovy duck, pekin duck and goose and one species of Colombiforms (pigeon). The chemerin abundance in egg white was significantly higher for the two species of duck compared to the other avian species tested even if the percentage of homology and identity sequence between duck RARRES2 gene and protein and these species was lower (Table 6). Probably because of phylogenetic distance, we were not able to detect the chemerin protein in human and mouse plasma or tissue (Figure 4) in agreement with the lack of chicken chemerin detection with anti-mammalian antibodies.

**TABLE 6:** Sequence homology and identity (%) for *RARRES2* gene and protein between chicken and two mammals (human and mouse) and various avian species.

*RARRES2*	*Gallus gallus*	
	Chicken	
	Gene (% homology)	Protein (% identity)
Human (*Homo Sapiens*)	56	37
Mouse (*Mus musculus*)	54	36
Turkey (*Meleagris gallopavo*)	94	90
Ring-necked pheasant (*Phasianus colchicus*)	93	92
Japanese quail (*Coturnix japonica*)	92	90
Guinea fowl (*Numida meleagris*)	91	89
Pekin duck (*Anas platyrhynchos*)	76	70
Swan goose (*Anser cygnoides*)	76	62
Rock Pigeon (*Columba livia*)	74	72

## Discussion

In the present study, we investigated for the first time plasma and egg white chemerin concentrations in seven hen breeds by using a home-made chicken chemerin monoclonal antibody and a new ELISA assay. We showed that chemerin egg white concentration was not correlated with chemerin plasma concentration and laying rate, whereas it was negatively correlated with total, egg white, and egg yolk weights, and fecundity and fertility.

We observed that chemerin concentrations in egg white were significantly higher than in blood plasma and there was no correlation between these two parameters. These data are in good agreement with our previous study (Estienne et al., 2022) showing that chemerin was 10 times more concentrated in egg white than in plasma. In the present study, we developed a specific chicken chemerin ELISA by using specific monoclonal chicken chemerin antibodies and recombinant chicken chemerin. The absence of link between plasma and egg white chemerin concentrations could originate from a local production of chemerin within the oviduct, more precisely at the magnum level, independently of chemerin in the blood circulation. Indeed, the oviduct’s magnum is the main site of egg white protein synthesis (Jeong et al., 2012). Furthermore, several studies demonstrated that chemerin mRNA and protein are found in the magnum of hen reproductive tract (Bílková et al., 2018; Estienne et al., 2022). Interestingly, we observed in each breed studied here, some hens with high or low chemerin protein levels in their egg white. However, the chemerin concentration in egg white as determined by both ELISA and Western-blot assays was relatively stable for eggs laid successively for 3, 4 or 5 days. These data suggest that egg white chemerin concentration could be used as a potential trait for genetic selection if these differences are conserved by the offsprings. Indeed, it could be interesting to study descendants of these hens over several generations in order to determine if these variations of chemerin concentrations in egg white are heritable. In the present study, we also observed significant differences of egg white chemerin between the seven breeds studied. For example, Fayoumi was one of the breeds with the higher egg white chemerin concentration as compared to OD and GAVORA breeds. Our results are also in a good agreement with a study showing that egg white protein abundance may differ between hens and between breeds (Wang et al., 2012).

Concerning egg performances, we showed that laying rates could be significantly different between breeds as well as the total egg, the albumen and the yolk weights. For example, Fayoumi hens laid fewer and smaller eggs probably because of their smaller body size as previously described (Khawaja et al., 2012; Kebede, E. 2017). In addition, we also established a link between egg white chemerin concentration and the egg parameters. We showed, for the first time, that egg white chemerin concentrations were negatively correlated with total egg, egg white and egg yolk weights. Studies have shown that egg weight is an important factor that influences the composition and the proportion of basic morphological elements, i.e., albumen, yolk, and shell (Bleu et al., 2019). Ayeni et al., 2018 reported that the eggs with the highest albumen content and the lowest yolk content showed worse hatchability results. Hagger et al., 1986 also found that the embryos that died during the entire incubation process were found in the heaviest eggs. Farooq et al., 2001 also observed a negative correlation between egg weight and hatchability rate. In addition, the body weight of hatched chicks depend on the weight of the eggs; hence, the larger the eggs, the heavier the chicks (Ramaphala and Mbajiorgu, 2013). An important indicator of chick quality is its length, which is highly positively correlated with the chick weight without the yolk sac. Thus, egg weight presents different physical and chemical qualities that affect hatchability and chick quality (Zakaria et al., 2009). Consequently, it is important to find biomarkers to genetically select hen laying eggs with optimal sizes. Furthermore, egg white and yolk are important in the food industry (Mine, Y. 2015). For example, foaming, emulsifying and gelling are functional properties of albumen.

In the present study, we showed that chemerin concentrations in the egg white were negatively correlated with two of the reproductive parameters of their mothers: fecundity and fertility. We have already shown a negative effect of chemerin on egg fertilization three days after artificial insemination (AI) with sperm pre-incubated with recombinant chicken chemerin (Estienne et al., 2020). At variance with these negative effects of chemerin in egg development, we reported in another previous study that *in-ovo* injections of anti-chemerin or anti-CMKLR1 antibodies to neutralize the chemerin system in egg white, increased embryo mortality (Estienne et al., 2022). In brief, too much chemerin appears deleterious but this hormone is indispensable for egg development probably because the chemerin system is involved in chicken embryo angiogenesis (Estienne et al., 2022). Taken together, data provide more evidence of a potential role of chemerin in egg performance and embryo development in chicken. However, in the present study, the hens’ plasma chemerin levels were not correlated with their reproductive parameters. In a previous study, we found that plasma chemerin concentrations were negatively correlated with egg hatchability in broiler breed hen (Mellouk et al., 2018b). This discrepancy could be due to the age and/or the breed of animals.Indeed, Mellouk et al. studied 39 weeks-old broiler hens whereas in our study we analysed data from laying hens at the end of the laying period (Mellouk et al., 2018b). Thus, plasma and egg white chemerin concentrations in broiler and laying hen during all their egg-laying period must be compared. It must be pointed out in this respect that chemerin plasma levels decreased during the laying period in turkeys (Diot et al., 2015).

## Conclusion

Taken together, our data show that chemerin concentration in egg white is negatively associated to egg weight and to hen fecundity and fertility parameters in chicken. Thus, chemerin could influence egg quality, chicken embryogenesis and chick quality. In consequence, determination of its level in egg white could be a potential trait for genetic selection. The improvement of reproductive rates and quality of chick would obviously have huge impacts for breeding companies thanks to the number of animals generated.Tixier-Boichard et al., 1994.

## Data Availability

The datasets presented in this study can be found in online repositories. The names of the repository/repositories and accession number(s) can be found in the article/Supplementary material.

## Abbreviations

BCA, bicinchoninic acid; BSA, Bovine Serum Albumin; CCRL2, C-C chemokine receptor-like 2; CMKLR1, Chemerin Chemokine-Like Receptor; IGF-1, Insulin-Like Growth Factor 1; ELISA, Enzyme-Linked ImmunoSorbent Assay; TIG2, Tazarotene-Induced Gene 2; EEM, Early Embryonic Mortality; GPR1, G protein receptor 1; LEM, Late Embryonic Mortality; RARRES2, Retinoic Acid Receptor Responder 2.
